# Flexible catalytic site conformations implicated in modulation of HIV-1 protease autoprocessing reactions

**DOI:** 10.1186/1742-4690-8-79

**Published:** 2011-10-10

**Authors:** Liangqun Huang, Yanfei Li, Chaoping Chen

**Affiliations:** 1 Department of Biochemistry and Molecular Biology, Colorado State University, Fort Collins, Colorado 80523-1870, USA

## Abstract

**Background:**

The HIV-1 protease is initially synthesized as part of the Gag-Pol polyprotein in the infected cell. Protease autoprocessing, by which the protease domain embedded in the precursor catalyzes essential cleavage reactions, leads to liberation of the free mature protease at the late stage of the replication cycle. To examine autoprocessing reactions in transfected mammalian cells, we previously described an assay using a fusion precursor consisting of the mature protease (PR) along with its upstream transframe region (p6*) sandwiched between GST and a small peptide epitope.

**Results:**

In this report, we studied two autoprocessing cleavage reactions, one between p6* and PR (the proximal site) and the other in the N-terminal region of p6* (the distal site) catalyzed by the embedded protease, using our cell-based assay. A fusion precursor carrying the NL4-3 derived protease cleaved both sites, whereas a precursor with a pseudo wild type protease preferentially autoprocessed the proximal site. Mutagenesis analysis demonstrated that several residues outside the active site (Q7, L33, N37, L63, C67 and H69) contributed to the differential substrate specificity. Furthermore, the cleavage reaction at the proximal site mediated by the embedded protease in precursors carrying different protease sequences or C-terminal fusion peptides displayed varied sensitivity to inhibition by darunavir, a catalytic site inhibitor. On the other hand, polypeptides such as a GCN4 motif, GFP, or hsp70 fused to the N-terminus of p6* had a minimal effect on darunavir inhibition of either cleavage reaction.

**Conclusions:**

Taken together, our data suggest that several non-active site residues and the C-terminal flanking peptides regulate embedded protease activity through modulation of the catalytic site conformation. The cell-based assay provides a sensitive tool to study protease autoprocessing reactions in mammalian cells.

## Background

HIV-1 protease (PR) is one of three virus-encoded enzymes essential for virus propagation and infectivity. The catalytic site of protease has been mapped to residue D25. Alteration of D25 to A, Y, H, or N completely abolishes enzymatic activity [1-4]. In the HIV-1 infected cell, the protease is initially synthesized as part of the Gag-Pol polyprotein precursor, within which the HIV-1 protease is flanked at the N-terminus by a transframe region named TFR or p6*, and at the C-terminus by the reverse transcriptase (RT) [2,5,6]. The regulated cleavage reactions, in which the Gag-Pol precursor is both the enzyme and substrate, lead to liberation of the free mature HIV-1 PR. This process is generally referred to as protease autoprocessing.

The released mature HIV-1 PR forms stable dimers and recognizes at least 10 different cleavage sites in the Gag and Gag-Pol polyproteins. Accurate and precise protease processing of these sites is absolutely required for the production of infectious progeny virions [7-13]. Therefore, the mature HIV-1 protease has been the primary target of anti-HIV drug development. In fact, unprecedented efforts from academic and industrial laboratories have made the mature HIV-1 protease one of the most-studied enzymes, as documented by numerous reports and reviews published over last 20 years [2,14-20]. These efforts have led to development of ten FDA-approved HIV-1 protease inhibitors for clinical applications. These inhibitors, however, all belong to the same mechanistic class--they are designed to bind to the catalytic site of the mature protease. Such single-mode inhibition is insufficient to completely suppress HIV-1 replication as drug resistant strains often emerge in patients under treatment. Therefore, novel therapeutic inhibitors with different mechanisms of action are urgently needed for the treatment of HIV-1 infection.

In distinct contrast to the extensive studies on the mature protease, the molecular and cellular mechanisms of HIV-1 protease autoprocessing are largely undefined. It is known that the protease domain embedded in the precursors is essential and sufficient to mediate autoprocessing because various precursors containing an active PR domain are able to release the mature protease when expressed *in vitro *[3,21], in *E. coli *[1,5,22-24], or in mammalian cells [8,25]. Of the two cleavage reactions that liberate the mature protease, the C-terminal cleavage reaction appears to be nonessential for virus replication. A mutation that blocks this cleavage site leads to production of PR-RT fusion enzymes, but the resulting viruses remain viable and infectious [26]. A transient intermediate consisting of the mature PR and a portion of the native C-terminal flanking sequence (the first 19 residues of RT) demonstrated proteolytic kinetics similar to the mature protease [27]. In addition, fusion of fluorescent proteins such as CFP and YFP to the C-terminus had no effect on protease dimerization and proteolytic activity [28]. In contrast, the N-terminal cleavage reaction is critical for liberation of the fully active mature protease. A p6*-PR fusion was unable to process most of the cleavage sites in the Gag polyprotein, leading to the production of noninfectious virions [29,30]. Removal of the p6* peptide was required for mature protease activity [23]. These studies have established the p6*-PR as a miniprecursor for autoprocessing characterization [5,23,24,31,32].

**Figure 1:**
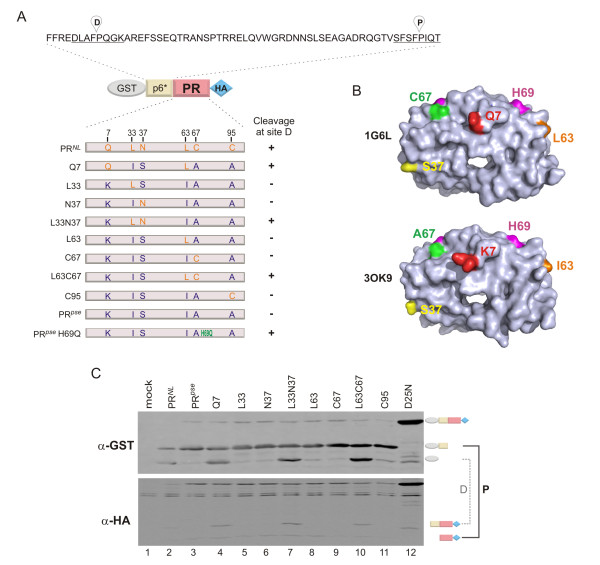
**Autoprocessing of fusion precursors with different protease sequences**. **(A) **Schematic diagram of the fusion precursors. The p6* sequence is derived from the NL4-3 strain, which contains two native cleavage sites, the distal (D) and proximal (P) sites, as indicated. **(B) **Space fill representation of two mature protease structures with the relevant surface amino acids highlighted in different colors. Top, an NL4-3 protease with residue S37 (the NL4-3 protease structure with N37 residue is not available); bottom, a pseudo wild type protease. **(C) **Autoprocessing of different precursors in transfected HEK293T cells. Post-nuclear cell lysates were prepared from cells transfected with plasmids encoding the indicated fusion precursors and subjected to western blot analysis. The blot was simultaneously probed using polyclonal rabbit anti-GST (upper) and mouse anti-HA (lower) primary antibodies, followed by IR700 goat anti-rabbit and IR800 goat anti-mouse secondary antibodies for concurrent visualization with an Odyssey infrared dual laser scanning unit. At right, the products released by the cleavage reaction at the D site are connected by a dotted line and those released by P site cleavage by a solid line.

Structural information on the embedded protease is currently unavailable in spite of more than 500 reported structures for the mature protease. Therefore, the mechanism by which the embedded protease mediates the autoprocessing cleavage reactions remains obscure. To facilitate examination of the cleavage reactions involved in protease autoprocessing, we previously engineered a fusion precursor consisting of a miniprecursor (p6*-PR) sandwiched between GST and a small peptide epitope (Figure 1A). GST was chosen as the N-terminal p6*-PR tag to stimulate precursor dimerization, which is believed to be important for the formation of a catalytic site based on the mature protease structure. The dissociation constant for GST dimerization is in the low nM range [33-35], and the GST C-termini are in close proximity in the crystallized GST dimer (PDB 3KMN). Because a protease antibody with high sensitivity is not available, a C-terminal peptide epitope was included to facilitate detection of the precursor substrate and processing products. The resulting fusion precursor effectively autoprocessed in *E. coli *and in transfected mammalian cells, and faithfully reproduced autoprocessing phenotypes observed in other systems [24,25]. This design provided an easy assay to study protease autoprocessing reactions inside cells, which differs from conventional studies in which proteolysis kinetics is characterized using purified mature proteases and synthetic peptide substrates in a test tube.

In this report, we examined two cleavage reactions involved in protease autoprocessing using protease inhibitors as a structural probe to gain insights into the catalytic site conformation of the protease under different contexts. Our data demonstrated that different protease constructs displayed varying sensitivities to inhibition by the currently available protease inhibitors, suggesting the existence of more than one catalytic site conformation. Interestingly, several surface residues far from the PR catalytic site, and residues adjacent to the PR C-terminus, also regulated the activity of the embedded protease involved in the autoprocessing cleavage reactions. Our data highlights a different catalytic mechanism driving liberation of the mature protease and provides a glimpse of the embedded protease as it functions during autoprocessing.

## Results and Discussion

### Different protease precursors demonstrate different cleavage preferences

A previously constructed fusion precursor contains two native cleavage sites, one between p6* and PR (the proximal (P) cleavage site) and the other at the N-terminal region of p6* (the distal (D) cleavage site) (Figure 1A). We tested two precursors with slightly different protease sequences [25]. One was derived from the NL4-3 strain, denoted as PR^*NL *^hereafter; the other was a pseudo wild type protease, PR^*pse*^, which was engineered to reduce protease self degradation (Q7K, L33I, and L63I) and protein aggregation mediated by thiol oxidation (C67A and C95A) for structural analysis of the mature protease *in vitro *[5,23,31]. When expressed in transfected mammalian cells, the mature PR^*pse *^is also self degraded [25]. There are a total of six residues that are different between PR^*NL *^and PR^*pse*^; all others are identical in these precursors (Figure 1A). Interestingly, the PR^*pse *^precursor predominantly autoprocessed the P site whereas the PR^*NL *^precursor autoprocessed both sites with a slight preference for D site cleavage (Figure 1C). Because the amino acid sequences at both cleavage sites are the same, we speculated the difference in substrate specificity is due to the difference in protease.

To identify which residues are attributed to the different substrate preference, we constructed a panel of PR^*pse *^precursors containing individual or combinatorial amino acid substitutions reflecting those present in PR^*NL *^(Figure 1A). Autoprocessing analysis of the resulting precursors demonstrated that a single Q7 mutation changed the cleavage preference from PR^*pse*^-like to PR^*NL*^-like, whereas a single C95 mutation did not. Also, we previously observed a PR^*NL*^-like autoprocessing phenomenon when single residue H69 was changed to Q, K, E or D in the PR^*pse *^backbone [25]. Single amino acid alterations at residues 33, 37, 63, or 67 did not change the cleavage preference, but the L33N37 and L63C67 double mutants displayed PR^*NL*^-like autoprocessing patterns. According to the crystal structure of the mature protease dimer, these residues are mostly surface exposed and far away from the active site (Figure 1B). These data suggest that multiple protease residues influence substrate preference of the embedded protease. Residues such as Q7 and H69 altered cleavage preferences by single amino acid mutation; others like L33N37 and L63C67 changed cleavage preferences by double mutation. These residue(s) or residue pair(s) are spread out on the mature protease surface, and they each seem to be sufficient to alter cleavage preferences. Our results are consistent with previous reports demonstrating that alterations in many non-active site residues are associated with evolution of drug resistant proteases causing formation of a catalytic site insensitive to a protease inhibitor yet active in proteolysis function [36-39].

It is very intriguing that different proteases display different preferences to the D and P cleavage sites. Since the cleavage sequences are identical in these fusion constructs, we suggest that different proteases have different catalytic sites that determine different substrate preferences. One could argue that different substrate accessibility might also be attributed to the observed difference. Although it is possible that the P site accessibility is altered by the adjacent PR, it is difficult to explain how the PR^*pse *^could render the D site noncleavable as it is separated from the protease by a flexible peptide (p6*). Therefore, we are inclined to suggest that different embedded proteases display different substrate preferences.

### The released proteases demonstrate different sensitivities to darunavir inhibition of self degradation

We next utilized darunavir, the most potent HIV-1 protease inhibitor, as a structural probe to examine the catalytic site conformation of various proteases. Darunavir binds to the catalytic site of the mature protease with low nanomolar affinity [40,41]. A previous study demonstrated that the most stable conformation of darunavir is very similar to that observed in the X-ray structure of darunavir in complex with the protease dimer [42]. Therefore, effective inhibition is expected if the catalytic site conformation readily accommodates darunavir; less suppression of proteolytic activity would be anticipated if the catalytic site is different from that reported in the mature protease structure.

**Figure 2:**
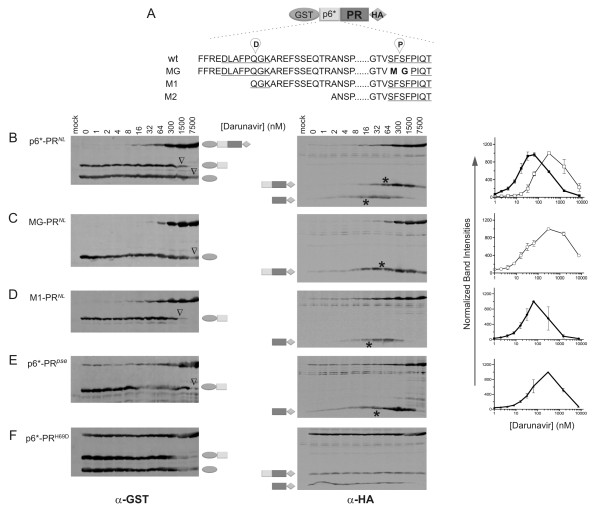
**Different fusion precursors respond differentially to darunavir inhibition**. All fusion precursors were derived from the GST-p6*-PR-HA construct. Differences in the p6* region are highlighted on the top **(A)**. The p6*-PR^*NL *^**(B) **contains the wild type NL4-3 protease sequence with two native cleavage sites. The P site was mutated in the MG-PR^*NL *^construct **(C)**; the D site was deleted in the M1- PR^*NL *^precursor **(D) **as previously described [25]. The p6*-PR^*pse *^precursor **(E) **is similar to the p6*-PR^*NL *^except that it contains the pseudo wild type protease sequence. The p6*-PR^H69D ^**(F) **precursor carries H69D point mutation in the p6*-PR^*NL *^backbone. Precursor autoprocessing in transfected HEK293T cells was examined in the presence of increasing concentrations of darunavir. Post-nuclear cell lysates were prepared at 30 h post transfection and subjected to western blot analysis. Each blot was simultaneous probed using polyclonal rabbit anti-GST (left) and mouse anti-HA (middle) primary antibodies, respectively, for dual visualization through two separate infrared channels. Open triangles indicate the apparent IC_50 _(left) for the cleavage reaction mediated by the embedded protease. Asterisks denote the IC_50 _for self degradation of the released PR-HA and p6*-PR-HA (middle). The band intensity of each PR-containing fragment was quantified, normalized to the highest pixel value (1000), and plotted against darunavir concentrations (right). Thick lines with solid symbols represent PR-HA produced by P site cleavage reaction; thin lines with open symbols represent p6*-PR-HA produced by D site cleavage reaction.

The wild type p6*-PR^*NL *^fusion precursor carries two native cleavage sites, D and P, respectively. To examine whether the cleavage reactions at these two sites interfere with each other, we engineered two fusion precursors to examine the individual reaction. The P site was mutated in the MG precursor, and the D site was deleted in the M1 precursor [25] (Figure 2A). Autoprocessing of the resulting precursors was essentially the same as observed with the wild type fusion precursor (Figure 2B-D), suggesting minimal interference between these two cleavage reactions in transfected cells. This also suggests that the secondary cleavage reactions mediated by the released proteases are minimal probably due to rapid diffusion and self degradation (below) in the cytoplasm of transfected cells.

We next examined effects of darunavir on the released proteases. In the absence of darunavir, the two PR-containing products, PR^*NL*^-HA and p6*-PR^*NL*^-HA, were not detectable likely due to rapid self degradation [43], while the GST-containing fragments were readily detectable (Figure 2B-D, left). In the presence of darunavir (8-300 nM), protease self degradation was inhibited such that the PR^*NL*^-HA and p6*-PR^*NL*^-HA fragments became detectable (Figure 2B-D, middle). Further increase in darunavir concentration reduced the amount of PR-containing products that were released. We interpreted this as a result of two relatively independent reactions. One reaction is self degradation of released PR^*NL*^-HA or p6*-PR^*NL*^-HA, the other is the cleavage reaction mediated by the embedded protease that liberate the autoprocessing products. Quantification of band intensities demonstrated the darunavir concentration where peak detection of the released protease was observed (Figure 2, right). At this concentration, the cleavage reaction mediated by the embedded protease was minimally suppressed as indicated by minimal accumulation of the full length precursor and effective production of GST-containing fragments. Accordingly, we were able to determine the IC_50 _to suppress self degradation (denoted by the asterisks).

The released PR^*pse*^-HA was less sensitive than PR^*NL*^-HA to darunavir inhibition of self degradation (Figure 2D&E), suggesting a difference in catalytic site conformation between these two mature proteases. Consistent with our observation, a slight difference in enzyme kinetics was reported between the mature PR^*pse *^and PR^*NL *^proteases when tested *in vitro *[23]. In addition, the p6*-PR^*NL*^-HA displayed a self degradation IC_50 _(~60 nM) approximately 6-fold higher than that for PR^*NL*^-HA (~10 nM), suggesting that they are not enzymatically identical. This is consistent with a previous report demonstrating that p6*-PR is incapable of processing many of the cleavage sites in the Gag polyprotein normally processed by the mature protease [30]. Taken together, our data indicate that the catalytic site conformation is modulated by different amino acid sequences in the mature protease (PR^*pse *^vs. PR^*NL*^) and also by the p6* peptide fused to the N-terminus of the mature protease (PR^*NL *^vs. p6*-PR^*NL*^).

It should be noted that the self degradation IC_50 _determined in our system is very similar to the IC_50 _identified for the mature protease activity in HIV-1 infected cells. Darunavir has an IC_50 _of ~5 nM to inhibit the production of p24 [40,41], whereas self degradation IC_50 _for PR^*NL*^-HA was ~10 nM. The slight difference might be attributed to varied protein concentrations. At least two factors might be attributed to this slight difference.  One factor is different readouts; the ~5 nM reflects the darunavir concentration required to achieve 50% inhibition of p24 production whereas the ~10 nM represents the concentration required to suppress the mature protease from self degradation by 50%.  The other factor is varied protein concentrations. Subsequently, more darunavir is required to suppress PR-HA self degradation in our cell-based assay. Nevertheless, our result is in agreement with the established darunavir IC_50_, further validating the utility of our assay for protease activity and autoprocessing analyses.

### The embedded protease is less sensitive than the mature protease to darunavir inhibition

With our assay system, the P and D site cleavage reactions are primarily catalyzed by the embedded protease as the released mature protease either is quickly self degraded or rapidly diffuses away in the absence of a Gag lattice as in a progeny virion. Darunavir binding to the embedded protease is expected to inhibit the cleavage reaction especially if the catalytic site conformation of the embedded protease is similar to that of the mature protease. According to the amount of the released GST protein, we estimated the apparent half maximal inhibition concentration (IC_50_) of the D site reaction to be ~7500 nM darunavir, as indicated by open triangles (Figure 2B and 2C, left). This is ~125-fold higher than the self degradation IC_50 _of the released p6*-PR^*NL*^-HA (~60 nM). The same trend was observed for the P site cleavage reaction, which had an apparent IC_50 _of ~1500 nM, *i.e*., ~150-fold higher than the self degradation IC_50 _of PR^*NL*^-HA (~10 nM). Additionally, the cleavage reaction IC_50 _of the embedded p6*-PR^*pse *^was ~7500 nM, whereas the self degradation IC_50 _for the released PR^*pse *^was ~40 nM (Figure 2E). It is intuitive to assume that the embedded protease and the free mature protease fold into similar structures with similar catalytic site conformations. However, our data demonstrated that the embedded protease is at least 100-fold less sensitive to darunavir inhibition than the corresponding released protease. This observation is consistent with a previous study reporting that an *in vitro *translated Gag-Pol precursor displayed significantly low sensitivity to ritonavir inhibition compared to the mature protease [21]. One might argue that this could be attributed to differences in dimerization ability of the embedded proteases as it is a prerequisite for the formation of a catalytic site. If it is the case, one should expect increased sensitivity to darunavir inhibition as the p6* peptide and darunavir treatment are mostly known to decrease protease dimerization [3,23,28,44,45], thus less functional catalytic sites are formed. In contrast, we observed low sensitivity, *i.e.*, active autoprocessing at high darunavir concentrations. Therefore, we interpreted that the low sensitivity to darunavir inhibition is due to the fact that the embedded protease has a catalytic site conformation different from that found in their corresponding mature protease.

Autoprocessing analysis of the PR^*NL *^H69D precursor further supported the idea that various catalytic site conformations exist (Figure 2F). The H69D mutation abolishes protease autoprocessing in the context of proviral constructs [46]. In our cell-based assay, the H69D fusion precursor autoprocessed both the D and P sites with low efficiency, as indicated by the presence of the full-length precursor in the lysate. Interestingly, the released PR-containing products were clearly detectable in the absence of darunavir, suggesting the released proteases were not degraded. Furthermore, darunavir treatment did not increase the amount of the released proteases, arguing for the existence of a catalytic site conformation that is resistant to darunavir inhibition. Similarly, autoprocessing reactions mediated by the embedded H69D PR were not suppressed by darunavir, suggesting that its catalytic site is not recognized by darunavir.

**Figure 3:**
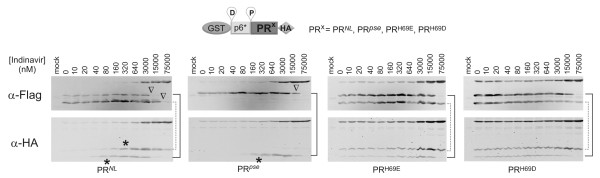
**Different fusion precursors respond differentially to indinavir inhibition**. All fusion precursors were derived from the GST-p6*-PR-HA construct. Differences in the PR region are listed on the top. Precursor autoprocessing in transfected HEK293T cells was examined in the presence of increasing indinavir concentrations. Post-nuclear cell lysates were prepared at 30 h post transfection and subjected to western blot analysis. Each sample was probed using mouse anti-Flag (top) or anti-HA (bottom) primary antibodies in parallel. Open triangles indicate the apparent IC_50 _for the embedded protease. Asterisks denote self degradation IC_50 _of the released PR-HA and p6*-PR-HA. The products released by the cleavage reaction at the D site are connected by a dotted line and those at the P site by a solid line.

We also tested autoprocessing of a few fusion precursors against indinavir, another well characterized protease inhibitor, and observed very similar results (Figure 3). The indinavir autoprocessing profiles were reminiscent to the darunavir ones, suggesting that our reported phenotypes herein are consistent with these two protease inhibitors. We conclude that the embedded and mature proteases with the same sequence display different catalytic site conformations, and that several protease residues modulate the catalytic site conformation in both the embedded and mature proteases.

### An indinavir resistant precursor is not resistant to darunavir inhibition

**Figure 4:**
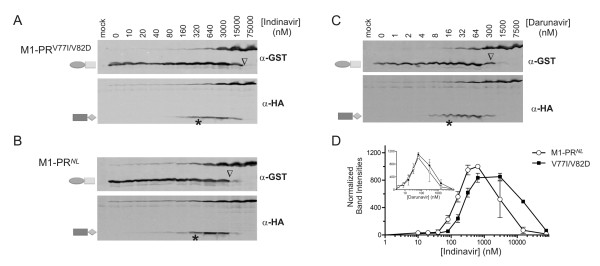
**An indinavir resistant precursor is not resistant to darunavir inhibition**. An indinavir resistant mutation (V77I/V82D) was introduced to the GST-M1-PR^*NL*^-HA backbone. Autoprocessing of the resulting construct with increasing concentrations of indinavir **(A) **or Darunavir **(C) **was compared to that of the parental precursor in the presence of indinavir **(B) **and darunavir (Figure 2D). Each blot was simultaneous probed and visualized through two infrared channels. The asterisks denote the self degradation IC_50 _of the released PR-HA; the open triangles denote the apparent IC_50 _of the cleavage reaction. The amounts of the released PR-HA from the parental (open circle) and mutant (solid square) precursor were quantified, normalized, and plotted against indinavir **(D) **or darunavir (D, inset) concentrations.

To further test the idea that different proteases have different catalytic site conformations, we constructed a fusion precursor carrying V77I and V82D. This double mutation was identified in a patient resistant to indinavir treatment [47]. The self degradation IC50 was ~150 nM and ~300 nM for the wild type and resistant mature protease, respectively (Figure 4). The mutant mature protease is thus ~two-fold less sensitive than the wild type protease to indinavir inhibition of self degradation, confirming a small difference in catalytic site conformation attributed to indinavir resistance. Consistent with our result, previous structural analysis revealed that the three-dimensional structures of the wild type protease or a multi-drug-resistant variant in complex with indinavir were only slightly different [48,49]. Interestingly, this subtle difference was not detected by darunavir; both wt and mutant mature proteases showed a self degradation IC_50 _of ~20 nM darunavir (Figure 4D, insert). Our data suggest that darunavir binds to the protease variants with similar affinity and kinetics despite the slight structural difference, providing evidence that darunavir is a better choice for treating drug experienced patients.

The cleavage reaction mediated by the embedded protease showed an apparent indinavir IC_50 _between 300 nM and 1500 nM for the wild type precursor (Figure 4B) and an apparent IC_50 _between 1500 nM and 7500 nM (Figure 4A) for the mutant precursor. Therefore, the mutation rendered greater drug resistance (~5-fold) to the cleavage reaction than to self degradation of the mature protease, suggesting that the mutation also causes a change in the catalytic site conformation of the embedded protease, which seems to contribute more to indinavir resistance. Once again, darunavir inhibited the cleavage reaction mediated by the control or mutant precursor to a similar extent (Figure 2D and 4C), confirming that darunavir is more effective and able to inhibit activity of an indinavir resistant protease. Collectively, our data illustrated that our assay is sensitive enough to detect subtle differences in the catalytic site between an indinavir resistant mutant (V77I/V82D) and its parental PR^*NL *^precursor, and that flexibility of catalytic site conformation is involved in regulation of both embedded and mature protease activities.

### C-terminal fusions moderately regulate the proximal site cleavage reaction

**Figure 5:**
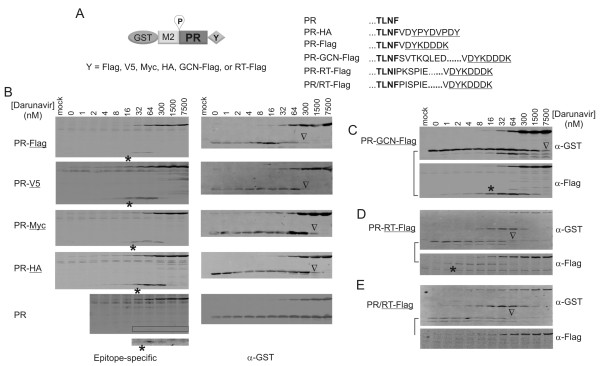
**Autoprocessing of precursors with different C-terminal tags**. All fusion precursors were derived from GST-M2-PR^*NL*^-Flag for specific examination of P site cleavage reaction. **(A) **The sequences at the junction between the PR and the C-terminal tag of some constructs are listed. **(B) **Autoprocessing of precursors carrying different small peptide epitopes or no tag were analyzed using a mouse epitope-specific antibody (Flag, Myc, HA), polyclonal rabbit anti-V5, or anti-PR as underlined at left, and GST primary antibodies for dual visualization. Because the signal detected by anti-PR antibody was weak, the boxed region was enhanced to show the mature protease (bottom). Autoprocessing of precursors fused to a GCN4 motif **(C) **or to reverse transcriptase (RT) in which the native cleavage site between PR and RT remained cleavable **(E) **or was mutated to uncleavable **(D) **were also compared. Asterisks indicate self degradation IC_50 _of the PR-containing product; open triangles indicate the apparent IC_50 _of the cleavage reaction. The expected products from P site cleavage reaction are connected by a solid line.

To examine whether different amino acid sequences fused to the C-terminus of PR influence protease autoprocessing, we engineered a panel of fusion precursors carrying different C-terminal epitopes (Figure 5A). A truncated version of M2 p6* (Figure 2A) was used to allow focused examination of the P site cleavage reaction. We also constructed GST-M2-PR, a precursor lacking any C-terminal epitope, to serve as a reference control. Self degradation IC_50 _of each the released proteases was between 16-32 nM darunavir, suggesting that small C-terminal peptide fusions have a minimal effect on the catalytic site conformation of the mature protease such that they were inhibited by darunavir similarly from self degradation. In contrast, different tags exhibited different effects on darunavir inhibition of the cleavage reaction catalyzed by the embedded protease. With the tagless precursor, the cleavage reaction was not suppressed even with 7500 nM darunavir. The apparent IC_50 _was ~1500 nM for the Myc- and HA-tagged precursors. The Flag and V5 peptides made the precursor more sensitive to darunavir inhibition (apparent IC_50 _~300 nM) than the HA and Myc epitopes, although there is no obvious correlation between the lengths or charge properties of the tags with this observed difference. Our data suggested that the embedded protease activity was modulated by different C-terminal tags, but self degradation of the mature protease after it was released from the precursor was not significantly affected by these tags. One possible cause for the increased sensitivity to darunavir could be that the C-terminal tags increased difficulty in precursor dimerization, and thus less active site were formed and less darunavir were required to suppress their catalytic activity. Alternatively, the C-terminal tag could directly modulate the enzymatic activity of the embedded protease by influencing the catalytic site conformation. Biophysical and structural analyses of these proteases are essential to definitely distinguish the possible causes.

To gain further insight into the effect of C-terminal flanking sequences on protease autoprocessing, we constructed more fusion precursors containing longer C-terminal fusions. The GCN4 dimerization motif derived from a yeast transcription factor [50-52] was directly fused to the C-terminus of the mature PR followed by Flag (Figure 5A). We chose the GCN4 motif to induce precursor dimerization from the C-terminus of the protease. The released PR-GCN4-Flag displayed a self degradation IC_50 _~20 nM darunavir, whereas the IC_50 _for the cleavage reaction was greater than 1500 nM. This was similar to the HA-tagged fusion precursor. We also detected additional fragments likely produced as a result of cleavage reactions at alternative sites at high darunavir concentrations, suggesting the existence of a catalytic conformation(s) induced by darunavir binding that process amino acid sequences not recognized at low darunavir concentrations. These data further confirmed that the embedded protease is much less sensitive to darunavir inhibition and its activity could be influenced by different C-terminal peptides that are adjacent to it.

In the Gag-Pol polyprotein precursor, the PR domain is followed by reverse transcriptase (RT), and there are reports suggesting a possible contribution of RT to regulation of protease activity [53,54]. We generated RT-containing precursors with the native cleavage site mutated (PR-RT) or kept unchanged (PR/RT) to examine their autoprocessing. The overall expression levels of the resulting precursors were much lower than the other precursors, likely because the reverse transcriptase coding sequence is not optimized for high levels of expression in transfected cells [55]. Nonetheless, self degradation IC_50 _of the released PR-RT-Flag was at the low nanomolar range (2-4 nM), and the cleavage reaction mediated by the embedded protease displayed an apparent IC_50 _of ~60 nM darunavir (Figure 5D). We were unable to detect the RT-Flag released from the precursor carrying the native cleavage site between PR and RT (Figure 5E), while the cleavage reaction demonstrated an IC_50 _of ~60 nM darunavir. The apparent high sensitivity to darunavir inhibition could be due to the low expression levels such that less darunavir were required to suppress the cleavage reaction. Alternatively, the catalytic site of the RT fusion precursors and the released PR-RT enzyme fits better for darunavir binding. Additional analyses using precursor constructs with compatible levels of expression would be necessary to further define the underlying cause of the decreased darunavir requirement. We also observed a possible alternative cleavage reaction at a site within the reverse transcriptase as indicated by a GST-containing fragment with an apparent MW greater than GST-M2-PR, whereas the other Flag-containing fragment was undetectable. This cleavage reaction occurred when darunavir concentrations were between 8 nM and 300 nM, suggesting a different catalytic conformation formed in this concentration range. This reaction was completely suppressed at high concentrations of darunavir. Taken together, these data suggest that different C-terminal flanking sequences could influence the proteolytic activity of the embedded protease by modulating the catalytic site conformation, revealing an additional dimension of protease complexity arising from plasticity of the embedded protease active site conformation.

### N-terminal fusions do not affect precursor autoprocessing

**Figure 6:**
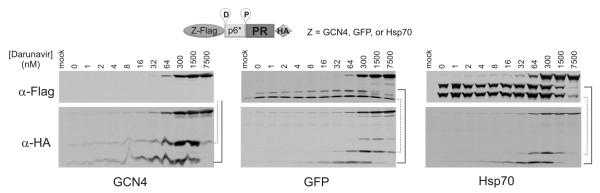
**Autoprocessing of precursors with different N-terminal tags**. The GST portion in the GST-p6*-PR-HA construct (Figure 2A) was replaced with a GCN motif (left), GFP (middle), or Hsp70 (right), respectively. The resulting precursors also contained a Flag peptide upstream of the D site. Blots were probed using either mouse anti-Flag (upper) or mouse anti-HA (lower) primary antibodies. The dotted lines connect the products expected from D site cleavage reaction and solid lines connect the products from P site cleavage reaction. The GCN4- containing products were too small to detect by SDS-PAGE.

To examine the role of N-terminal fusions on precursor autoprocessing, we replaced the GST with a GCN4 motif, GFP or hsp70. Both the D and P cleavage sites were included in these constructs and a Flag peptide was in-frame fused to the motif/proteins to simplify detection. All three fusion precursors were autoprocessed effectively in the absence of darunavir, as indicated by the disappearance of the fusion precursor (Figure 6). The released fragments GCN4-Flag and GCN4-Flag-p6* were too small to detect by SDS-PAGE. The released PR-HA and p6*-PR-HA showed self degradation IC_50_s that were very similar to the corresponding values observed for the fragments released from the GST fusion precursors (Figure 2A). At high darunavir concentrations, the GFP precursor released extra fragments which is likely due to a cleavage reaction at an alternative site. The cleavage reactions at the D or P sites catalyzed by the embedded protease also displayed similar response profiles to darunavir inhibition as the GST fusion precursor (Figure 2A). This data suggested that fusions at the N-terminus of p6* do not significantly influence the catalytic site conformation of the embedded protease, likely because they are separated from the protease domain by a long and flexible peptide (p6*).

## Conclusions

In this study, we studied two proteolysis reactions involved in HIV-1 protease autoprocessing in transfected cells expressing engineered fusion precursors. Protease inhibitors designed to specifically bind to the catalytic site of the mature protease were used as structural probes to examine the catalytic site conformation. Our analyses demonstrated that both the protease sequence and the protease context (free mature vs embedded with flanking peptides) affect the catalytic site conformation, which in turn alter the response sensitivity to inhibition by catalytic site inhibitors. Several non-active site residues as well as residues flanking the protease also contributed to modulation of catalytic site conformation. Our results are consistent with extensive structural studies that demonstrate multiple slightly different catalytic site conformations in mature protease as a molecular basis for the evolution of drug resistant strains. The advantage of our assay is simplicity without a compromise of sensitivity, allowing for broad application in the examination of the protease autoprocessing mechanism and/or in the identification and characterization of novel anti-HIV drugs. Our results also imply that novel inhibitors targeting important surface residues may be developed as alternative therapeutic agents. An encouraging report along this vein identified a novel inhibitor from combinatorial libraries that suppresses HIV-1 protease activity likely through binding to a groove outside of the catalytic site [56]. Identification of such inhibitors that interfere with protease activity via modes of action different from the current protease inhibitors will provide new promises for future development of therapies to defeat the spread of HIV-1.

## Methods

### DNA mutagenesis

Construction of plasmids encoding GST-p6*-PR^*NL*^-HA, GST-p6*-PR^*pse*^-HA, GST-p6*-PR^*pse *^D25N-HA, GST-p6*-PR^*NL*^H69D-HA, GST-M1-PR^*NL*^-HA, and GST-M2-PR^*NL*^-HA was previously described [25]. Additional mutations described herein were introduced into the indicated plasmids by standard PCR-mediated mutagenesis and cloning procedures. Mutations Q7, L33, N37, L33N37, L63, C67, L63C67 and C95 were individually introduced into the GST-P6*-PR^*pse*^-HA expression plasmid. The GST-p6*-MG-PR^*NL*^-HA construct was generated by mutating the amino acids at the P site (from TVSFSF⇓PQIT to TVMG⇓PQIT) to block proteolytic cleavage. The indinavir resistant mutation V77V82 was cloned into the plasmid encoding GST-M1-PR^*NL*^-HA. To generate fusion precursors with various C-terminal sequences, the Flag tag coding sequence in GST-M2- M2-PR^*NL*^-Flag was replaced by sequences encoding the indicated epitopes, motif or proteins. Similarly, the GST coding sequence in the GST-Flag-PR^*NL*^-HA expression plasmid was replaced with GCN4, GFP, or Hsp70 [57] encoding sequence for expression of fusion precursors containing different N-terminal sequences. All the constructs were verified by sequencing analysis and detailed plasmid information is available upon request.

### Cell culture and transfection

HEK293T cells (ATCC, Manassas, VA) were maintained in DMEM containing 10% fetal bovine serum and penicillin/streptomycin (0.6% Penicillin G sodium salt, 1.0% Streptomycin sulfate, 0.85% NaCl). Plasmid DNA was transfected into HEK293T cells using calcium phosphate as previously described [25,46]. Briefly, HEK293T cells were seeded in 12-well plates the day before transfection to achieve 50~60% confluence at the time of transfection. About 1 h prior to transfection, chloroquine was added into each well to a final concentration of 25 μM. A total of 0.5 μg plasmid DNA in 65.7 μl H_2_O was mixed with 9.3 μl of 2 M CaCl_2_. Then 75 μl of 2xHBS (50 mM Hepes, pH7.04~7.05, 10 mM KCl, 12 mM Dextrose, 280 mM NaCl, 1.5 mM Na_2_HPO_4_) was added to the DNA-Ca mixture followed by gentle agitation. The resulting mixture was directly added drop-wise to each well. Protease inhibitors darunavir (NIH AIDS research and reference program, Cat# 8145) and indinavir (NIH AIDS research and reference program, Cat# 11447) were dissolved in autoclaved H_2_O to generate 250 μM darunavir and 10 mM indinavir stock solutions, respectively; these were stored at -20°C. darunavir or indinavir were then diluted and added to cells directly after transfection at the indicated working concentration. After 7-8 h incubation at 37°C, 5% CO_2_, the transfection medium was replaced with fresh chloroquine-free medium containing the corresponding protease inhibitors. At 30 h post-transfection, cells were washed once with PBS and lysed with 100 μl lysis buffer A (Tris-HCl, pH8.0, 150 mM NaCl, 1% sodium deoxycholate, and 1% Triton X-100, and protease inhibitor cocktail). Cell lysates were clarified by brief centrifugation at 20800 × *g *for 2 minutes and stored at -20°C for further western blot analysis.

### Western blotting and quantification

Primary antibodies used in this study include polyclonal rabbit anti-GST (a kind gift from Dr. Santiago DiPietro, Colorado State University), anti-V5 (Rockland, Cat# 600-401-378), anti-HIV1 protease (NIH AIDS research and reference program, Cat# 4105); mouse monoclonal anti-HA (Sigma, Cat# H9658), anti-flag (Sigma, Cat# F1804), and anti-myc (purified from culture medium of hybridoma cells, ATCC, Cat# CRL-1729). Secondary antibodies included IR700 goat anti-rabbit (Rockland, Cat# 611-130-122) and IR800 goat anti-mouse (Rockland, Cat# 610-132-121). For protein detection, about 1/6 of the cell lysate from one well of a 12 well plate was subjected to SDS-PAGE followed by western blot analysis. The blots were probed simultaneously or separately with the corresponding primary and secondary antibodies, and visualized with an Odyssey infrared dual laser scanning unit (LI-COR Biotechnology, Lincoln, Nebraska). To reduce background noise, the rabbit anti-HIV protease antibody was first incubated with cell lysates from untransfected 293T cells that had been resolved by SDS-PAGE and transferred onto a PVDF membrane.

For quantification of protein intensity, Western blot images captured by an Odyssey infrared dual laser scanning unit were analyzed using TotalLab software (Nonlinear Dynamics Inc.). The total pixel volume of each band was quantified and normalized; the highest value was arbitrarily set at 1000.

## Competing interests

Colorado State University has filed for patent protection of this work.

## Authors' contributions

CC designed the experiments and wrote the manuscript. LH constructed most of the plasmids used in this study and performed 293T transfections and Western blot analyses. YL made the plasmids used in Figures 1 and 4 and performed several transfections and the subsequent Western blot analyses. All authors read and approved the final manuscript.
